# Dynamic response landscape of immune cells identified immune dysfunction which predicts disease progression in COVID-19 infected patients

**DOI:** 10.7150/ijbs.71163

**Published:** 2022-04-24

**Authors:** Xiangyu Deng, Yao Zhang, Meiqi Li, Xiaolong Tang, Jing Shen, Yu Chen, Yueshui Zhao, Qinglian Wen, Xu Wu, Mingxing Li, Jing Li, Wenping Xu, Wanping Li, Zhangang Xiao, Deqiang Xian, Fukuan Du

**Affiliations:** 1 Laboratory of Molecular Pharmacology, Department of Pharmacology, School of Pharmacy, Southwest Medical University, Sichuan Luzhou 646600, China; 2 Cell Therapy & Cell Drugs of Luzhou Key Laboratory, Sichuan Luzhou, 646000, China; 3 South Sichuan Institute of Translational Medicine, Sichuan Luzhou 646600, China; 4 Department of Oncology, Affiliated Hospital of Southwest Medical University, Sichuan Luzhou 646600, China; 5 Department of Oncology and Hematology, Hospital (T.C.M) Affiliated to Southwest Medical University, Sichuan Luzhou 646600, China; 6 Luzhou Center for Disease Control and Prevention, Sichuan Luzhou 646600, China

**Keywords:** SARS-CoV-2, COVID-19, T Cells, NK Cells, Single Cell RNA-seq

## Abstract

During the development of COVID-19 caused by SARS-CoV-2 infection from mild disease to severe disease, it can trigger a series of complications and stimulate a strong cellular and humoral immune response. However, the precise identification of blood immune cell response dynamics and the relevance to disease progression in COVID-19 patients remains unclear. We propose for the first time to use changes in cell numbers to establish new subgroups, which were divided into four groups: first from high to low cell number (H_L_Group), first from low to high (L_H_Group), continuously high (H_Group), and continuously low (L_Group). It was found that in the course of disease development. In the T cell subgroup, the immune response is mainly concentrated in the H_L_Group cell type, and the complications are mainly in the L_H_Group cell type. In the NK cell subgroup, the moderate patients are mainly related to cellular immunity, and the severe patients are mainly caused by the disease, while severe patients are mainly related to complications caused by diseases. Our study provides a dynamic response of immune cells in human blood during SARS-CoV-2 infection and the first subgroup analysis using dynamic changes in cell numbers, providing a new reference for clinical treatment of COVID-19.

## Introduction

Coronavirus disease 2019 (COVID-19) was induced by the severe acute respiratory syndrome coronavirus-2 (SARS-COV-2). COVID-19 is extremely contagious and spreads rapidly at home and abroad^1^. It is a disease mainly characterized by fever, fatigue, cough, and pneumonia^2^. SARS-CoV-2 belongs to the genus β-coronavirus, which consists of a genome of approximately 30 thousand bases and is an enveloped single-stranded RNA virus^3^, ^4^. This disease currently poses a great challenge to humans. So far, there is no highly effective treatment method for COVID-19, and the main pathogenesis of COVID-19 in humans has not been understood^5^. It has become an urgent need to understand the pathogenesis of COVID-19 for clinical treatment.

SARS-CoV-2 is highly pathogenic and poses a great threat to public health. SARS-CoV-2 infection activates both innately and adaptive immune responses^6^. The transition between innate and adaptive immune responses is critical to the clinical progression of SARS-CoV-2 infection^7^. The early immune response mainly plays a protective role, while the dysregulation and aggravation of the inflammatory response cannot clear the virus and cause the disease to worsen. The accumulation of pro-inflammatory cytokines, lymphopenia, and abnormal T cell response provide evidence that COVID-19 may be an immune-related disease^8^.

We use single-cell sequencing analysis to study how the dynamic changes in the number of immune cells in the blood of COVID-19 patients and healthy individuals affect the progression of COVID-19 patients. In previous studies, single-cell analysis of COVID-19 was based on the overall level of immune cells, which led to the average number of cells. Our study found that the changes in the number of immune cells in the course of disease were caused by the changes in the number of cell subgroups. It is worth noting that the immune response in the T cell subgroup is mainly concentrated in the H_L_Group cell type, and the complications caused by the virus are mainly in the L_H_Group cell type. In the NK cell subgroup, the immune response is mainly concentrated in the front end of the disease progression, and the complications caused by viruses were mainly in the back end of the disease progression. This study provides us with a better understanding of the specific cell subgroups that contribute to the progression of the disease, which will bring us closer to identifying possible interventions for the treatment of COVID-19, especially those with severe complications.

## Results

**Figure 1:**
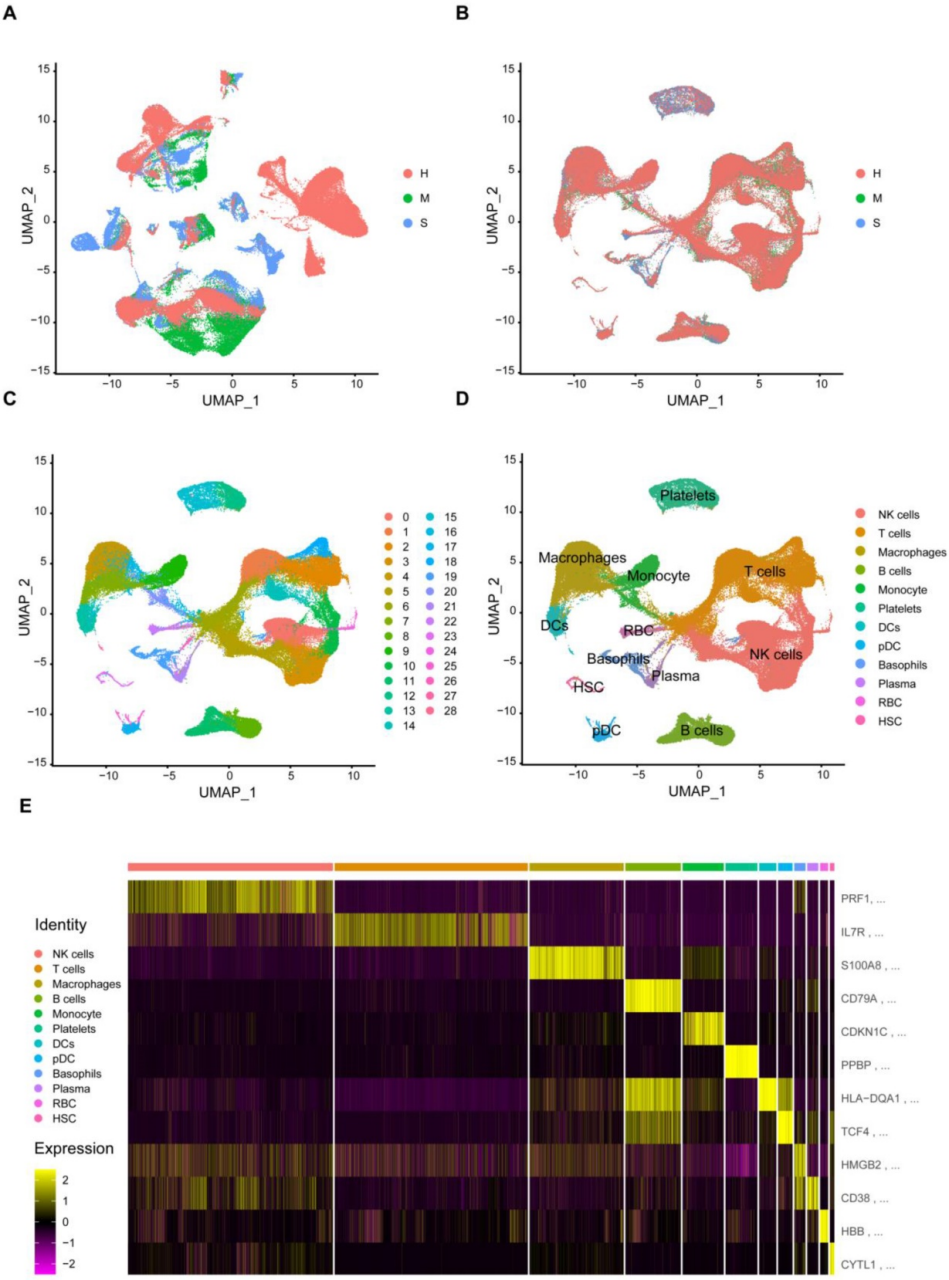
** Single-Cell Map of COVID-19 patients and controls.** (A) UMAP shows all sample types. Healthy (H), moderate (M), and severe (S) patients were all samples before the removal of batch effects. (B) UMAP shows all sample types. Healthy (H), moderate (M), and severe (S) patients after removal of batch effects for all samples. (C) Clustering of single cells in UMAP space, showing 29 clusters (clusters 0-28). (D) Cell populations were identified. The UMAP projections of 136014 single cells from healthy (n = 11), moderate (n = 8), and severe (n = 7) samples show 12 clusters with their respective markers. Each point corresponds to a cell and is colored according to the cell type. (E) Heat map showing the expression levels of specific marker genes according to each cluster.

Identification of cell types in the blood of healthy individuals and patients with COVID-19. To assess the progress of immune cells in COVID-19 patient blood, we first analyzed published single-cell sequencing data sets from COVID-19 patient blood samples^9^-^11^. After data filtering, 136,014 cells passed the standard quality control and were used for subsequent analysis. Next, we show the number of genes, unique molecular identifiers (UMIs), and the percentage of mitochondrial genes in the single-cell data (Figure S1). First, we performed an unsupervised cluster analysis on the entire data set to eliminate batch effects across different individuals (Figure 1A and 1B). We use ElbowPlot to determine the dimension of the data set. From Supplementary Figure 2A, we can see that the change tends to be flat after PC=30. We use PC1 to 30 for clustering. Through unified manifold approximation and projection (UMAP) and marker gene analysis, a total of 136,014 blood cells were identified. Based on the results of UMAP, we identified 28 main cell clusters, and none of them came from a single person (Figure 1C). According to the expression of canonical lineage markers and other genes up-regulated in each cluster (Figure S2B-S2D). We have identified cell types, including NK cells (PRF1, GNLY, NKG7, CCL5, KLRD1), T cells (IL7R, CD3E, CD69, CD8B, CD3D), Macrophages (S100A8, S100A9, FCN1, CD14, FCN1), B cells (CD79A, MS4A1, CD19, CD79A, CD79B), Monocytes (CDKNC1, NEAT1, FCGR3A, MS4A7 ), Platelets (PPBP, PF4), DCs (HLA-DQA1, FCER1A, IRF8, CLEC9A), pDCs (TCF4, ITM2C, LILRA4, CLEC4C), Basophils (HMGB2, CD38), Plasma cell (CD38, IGKC), RBC (HBB, HBA2, HBA2), HSC (CYTL1, GATA2, CDK6) (Figure 1D and 1E), For marker genes, the expression value in each cell located in UMAP (Figure 1E).

**Figure 2:**
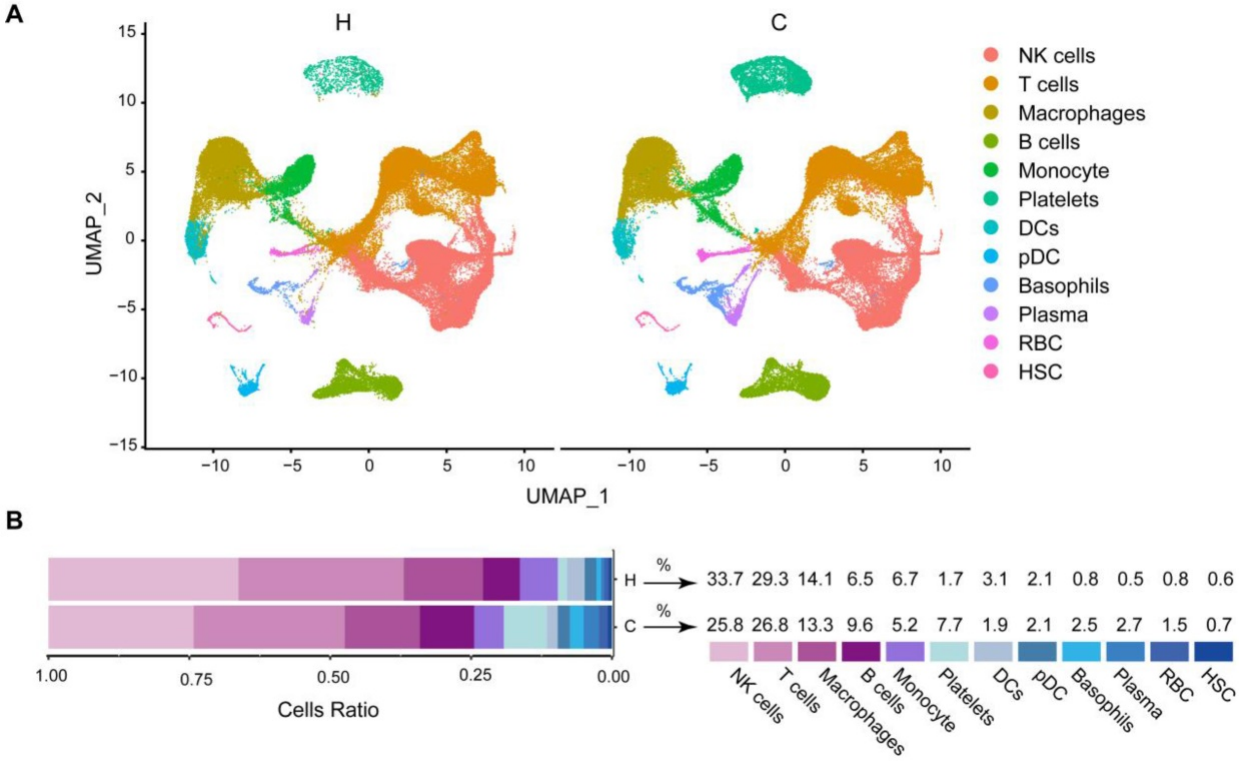
** Comparison of basic characteristics between healthy and covid-19 patient groups.** (A) UMAP projection of healthy (H), moderate and severe (C) patient samples in different cells. Each point corresponds to an individual cell, colored according to cell type. (B) Frequency of sample types in each cell type. The bar chart is colored according to the sample type. The average proportion of each cell type in healthy (H), moderate and severe (C) patient samples.

Comparison of basic characteristics between healthy people and COVID-19 patients. In order to determine the basic characteristics of immune cells in the blood of healthy people and COVID-19 patients, we analyzed the changes in different cell types in healthy people and COVID-19 patients. In healthy people (H) and COVID-19 patients (C), we found a decrease in NK cells, T cells, macrophages, monocytes, and DCs. B cells, Platelets, Basophils, Plasma, RBC, and HSC cells increased. The number of pDC cells does not change (Figure 2A and 2B). Notably, COVID-19 patients have the highest proportion of NK cells and T cells (Figure 2B). In summary, our results show that NK cells and T cells make up the largest fraction and that the number of cells has been decreasing in COVID-19 patients. In order to have a more precise understanding of the role of immune cells in disease progression in COVID-19 patients, we next divided COVID-19 patients into mild and severe patients for further study.

**Figure 3:**
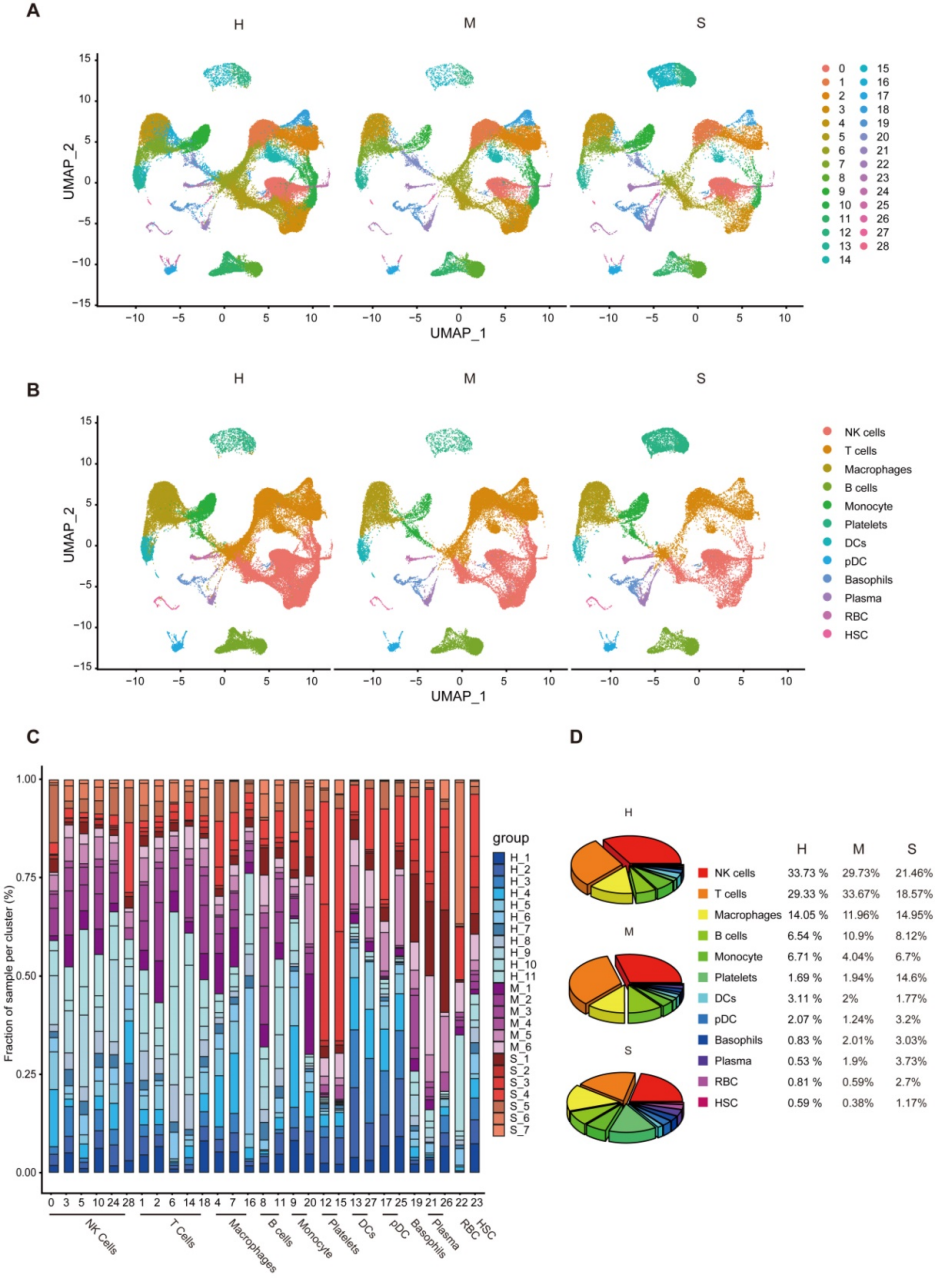
** Dynamic composition of immune cells during COVID-19 infection.** (A) Display of healthy (n = 11), moderate (n = 8), and severe (n = 7) patient samples in different clusters of UMAP projection. Each point corresponds to an individual cell, colored according to cell type. (B) UMAP projection of healthy (n = 11), moderate (n = 8), and severe (n = 7) patient samples in different cells. Each point corresponds to an individual cell, colored according to cell type. (C) Frequency of sample types in each cell type and different clusters. The bar chart is colored according to the sample type. (D) The average proportion of each cell type in healthy (n = 11), moderate (n = 8), and severe (n = 7) patient samples.

**Overview of immune cells in the blood of patients with severe and mild COVID-19 compared with healthy subjects.** In order to assess the blood immune system status of COVID-19 patients, we analyzed the changes of immune cells in the blood of COVID-19 patients in different clusters, and the changes in the number of cells in healthy people, moderate and severe patients. We found that the number of cells changed in different clusters as the disease progressed (Figure 3A). We further analyzed the changes in the progression of COVID-19 patients in different cell types. The UMAP chart shows that NK cells and T cells have obvious changes during the progression of the disease (Figure 3B). Next, we found that T cells and B cells increased first and then decreased in healthy individuals, moderate and severe COVID-19 patients. Macrophages, monocytes, pDC, RBC, and HSC cells are in a state of decreasing first and then increasing. Platelets, Basophils, and Plasma cells have been increasing. NK cells and DCs have been decreasing (Figure 3C and 3D). It is worth noting that COVID-19 patients had the highest proportion of NK and T cells (Figure 3C and 3D). In general, our results show that the number of NK cells in healthy individuals, moderate and severe COVID-19 patients have been decreasing, while T cells are in a state of first increasing and then decreasing, and the immune cell composition varies between patients.

**Figure 4:**
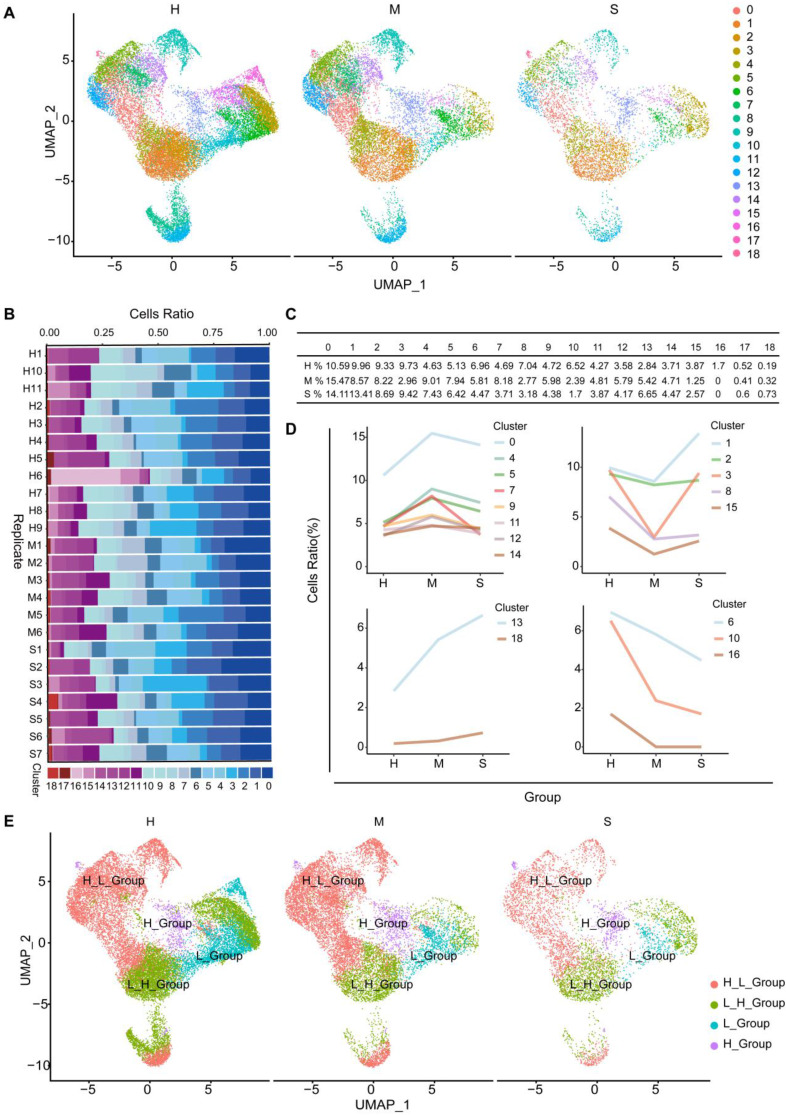
** Subgroup composition and dynamic changes of T cells.** (A) UMAP projection in T cell subgroups shows healthy (n = 11), moderate (n = 8), and severe (n = 7) patient samples in different clusters. (B) The bar chart shows the composition of T cell clusters at the single sample level, colored according to different cluster types. (C) The proportion of each T cell cluster obtained from healthy (n = 11), moderate (n = 8), and severe (n = 7) patient samples. (D) The line graph shows the changes in the number of healthy (n = 11), moderate (n = 8), and severe (n = 7) patient cells. (E) UMAP projection shows the renaming of T cell subgroups.

**Figure 5:**
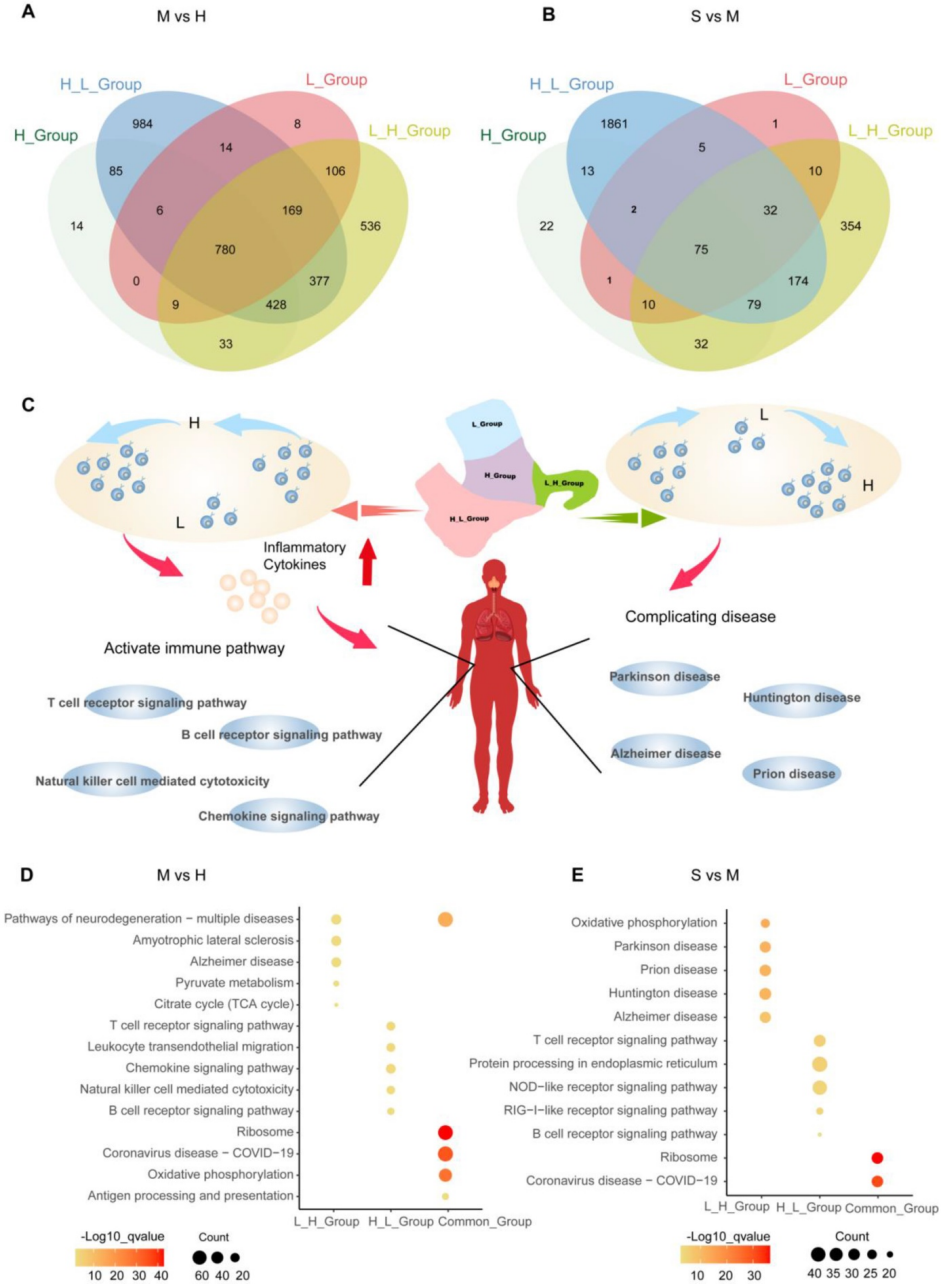
** T cell response pattern during COVID-19 infection.** (A) Moderate COVID-19 patients have a gene overlap in T cell subgroups (H_L_Group, L_H_Group, H_Group, and L_Group). (B) Severe COVID-19 patients have a gene overlap in T cell subgroups (H_L_Group, L_H_Group, H_Group, and L_Group). (C) Summarize the impact on the H_L_Group and L_H_Group subgroups in the process of COVID-19 infection. (D) In T cell subgroups, moderate patients (M) were compared with healthy controls (H). H_L_Group specific genes, L_H_Group specific genes, and genes common to all T cell subgroups (Common_Group) KEGG pathway enrichment analysis, node size represents the number of genes, and color intensity represents -log10 q-value. (E) In T cell subgroups, severe patients (S) were compared with moderate patients (M). H_L_Group specific genes, L_H_Group specific genes, and genes common to all T cell subgroups (Common_Group) KEGG pathway enrichment analysis, node size represents the number of genes, and color intensity represents -log10 q-value.

**Characteristics of T cell subgroups and their responses in the blood of COVID-19 patients.** We use UMAP to divide T cell subgroups into 19 different clusters (Figure 4A). To further understand the changes of T cells in the onset of COVID-19 patients, we found that the proportion of cells in the 19 clusters at different stages is different (Figure 4B and 4C). We divided them into four different subgroups according to the type of response to the virus. The number of cells first from high to low cell number (H_L_Group), first from low to high (L_H_Group), continuously high (H_Group), and continuously low (L_Group) (Figure 4D and 4E). In order to find out whether the pathways are heterogeneous in different T cell subgroups, we intersected the different genes of each cell subgroup in healthy individuals, moderate and severe patients to find out the unique genes (Table S1). We obtained that when healthy individuals developed the moderate disease, H_L_Group had 984 genes and L_H_Group had 106 genes. H_Group has 14 genes, L_Group has 8 genes and Common_Group has 780 genes (Figure 5A). We performed Gene Ontology (GO) enrichment analysis and Kyoto Encyclopedia of Genes and Genomes (KEGG) enrichment analysis for these genes. From healthy development to moderate disease, GO analysis showed that H_L_Group was mainly enriched in regulation of mRNA metabolic process, RNA splicing; L_H_Group is mainly enriched in regulation of mRNA metabolic process, RNA splicing; Common_Group was mainly enriched in protein targeting to ER, establishment of protein localization to endoplasmic reticulum (Figure S4A-S4C; Table S2).

In addition, KEGG enrichment analysis revealed that during the moderate infection stage, we found that SARS-CoV-2 infection shared with other viral infection pathways, for example, Human immunodeficiency virus 1 infection, Human cytomegalovirus infection, Human T-cell leukemia virus 1 infection, Epstein-Barr virus infection, Influenza A, Viral myocarditis, Measles, Kaposi sarcoma-associated herpesvirus infection, Prion disease. In addition, in our analysis, SARS-CoV-2 infection was found to be the same as bacterial, parasitic, and protozoan disease pathways. Related to Shigellosis, Yersinia infection, Vibrio cholerae infection, Salmonella infection, Bacterial invasion of epithelial cells. In addition, we found that SARS-CoV-2 is closely associated with Pathways of neurodegeneration-multiple diseases, and shares pathways with other diseases. The pathways associated with SARS-CoV-2 infection also include the T cell receptor signaling pathway, Regulation of actin cytoskeleton, Ubiquitin mediated proteolysis, Leukocyte transendothelial migration, Parathyroid hormone synthesis, secretion and action, Long-term potentiation, FOXO signaling pathway, Thyroid hormone signaling pathway, Apoptosis, Gap junction, Endocytosis, GnRH signaling pathway, Oxytocin signaling pathway, Th17 cell differentiation, Natural killer cell mediated cytotoxicity, cGMP-PKG signaling pathway, Insulin signaling pathway, Endocrine and other factor-regulated calcium reabsorption, Inflammatory mediator regulation of TRP channels, Circadian rhythm, VEGF signaling pathway, B cell receptor signaling pathway, Chemokine signaling pathway, Endocytosis, NOD-like receptor signaling pathway, Autophagy-animal, Necroptosis, Proteasome, Pyruvate metabolism, Citrate cycle (TCA cycle), Propanoate metabolism, Ribosome, Oxidative phosphorylation, Thermogenesis, Spliceosome, Focal adhesion, Protein export, RNA transport, Protein processing in endoplasmic reticulum, Cellular senescence, RNA degradation, Antigen processing and presentation (Figure 5C and 5D; Table S3).

In order to further investigate the role of specific genes in each subgroup of COVID-19 patients as they progressed from moderate to severe, we applied the same method above to obtain 1861 genes in H_L_Group, 354 genes in L_H_Group, 1 gene in H_Group, 22 genes in L_Group, and 75 genes in Common_Group (Figure 5B). These genes were analyzed by GO and KEGG enrichment. GO analysis shows that H_L_Group is mainly enriched in ribonucleoprotein complex biogenesis, regulation of chromosome organization; L_H_Group was mainly enriched in oxidative phosphorylation, mitochondrial ATP synthesis coupled electron transport; Common_Group is mainly enriched in SRP-dependent cotranslational protein targeting to membrane, cotranslational protein targeting to membrane (Figure S4D-S4F; Table S4). In addition, KEGG enrichment analysis found that Common_Group was mainly enriched in Ribosome, Coronavirus disease-COVID-19; H_L_Group was mainly enriched in Pathways of neurodegeneration-multiple diseases, Alzheimer disease; L_H_Group was mainly enriched in Pathways of neurodegeneration-multiple diseases, Alzheimer disease. Enriched in Huntington disease, Pathways of neurodegeneration-multiple diseases (Figure 5C and 5E; Table S5).

**Figure 6:**
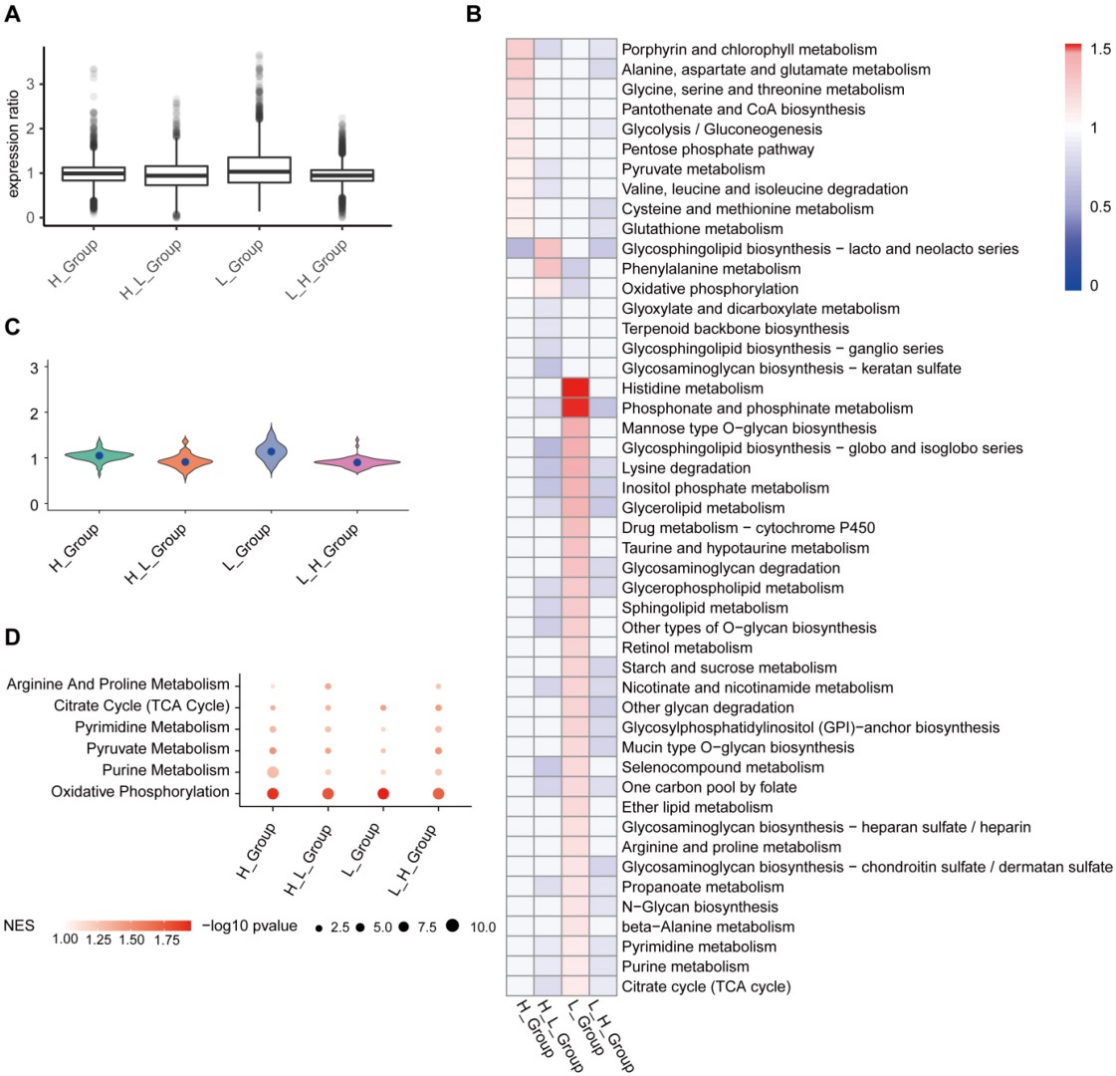
** T cell subgroups were significantly up-regulated in oxidative phosphorylation.** (A) The Deconvolution method was used to standardize the single-cell data, and the results showed that the expression ratio after the correction was more consistent. (B) Metabolic pathway activity in T cell subgroups. (C) Distribution of metabolic pathway activity in T cell subgroups. (D) Major metabolic pathways are enriched in all subgroups. The node size is -log10 p-value. Color intensity indicates the enrichment score (NES) after correction.

In order to get the metabolic activity of cells, we use single-cell expression data as input data, the data matrix we downloaded from GSEA metabolic gene sets (https://www.gsea-msigdb.org/gsea/index.jsp). In addition, we used the scimpute package to assign data missing values and used the Deconvolution method to standardize single-cell data (Figure 6A). H_Group has high activity in Porphyrin and chlorophyll metabolism, Alanine, aspartate and glutamate metabolism; H_L_Group has high activity in Glycosphingolipid biosynthesis-lacto and neolacto series, Phenylalanine metabolism; in L_Group, Histidine metabolism, Phosphonate and phosphinate metabolism higher activity (Figure 6B). On the whole, H_L_Group showed high activity in the whole metabolic activity analysis (Figure 6B and 6C). GSEA results showed that T cell subgroups were significantly up-regulated in oxidative phosphorylation (Figure 6D).

**Figure 7:**
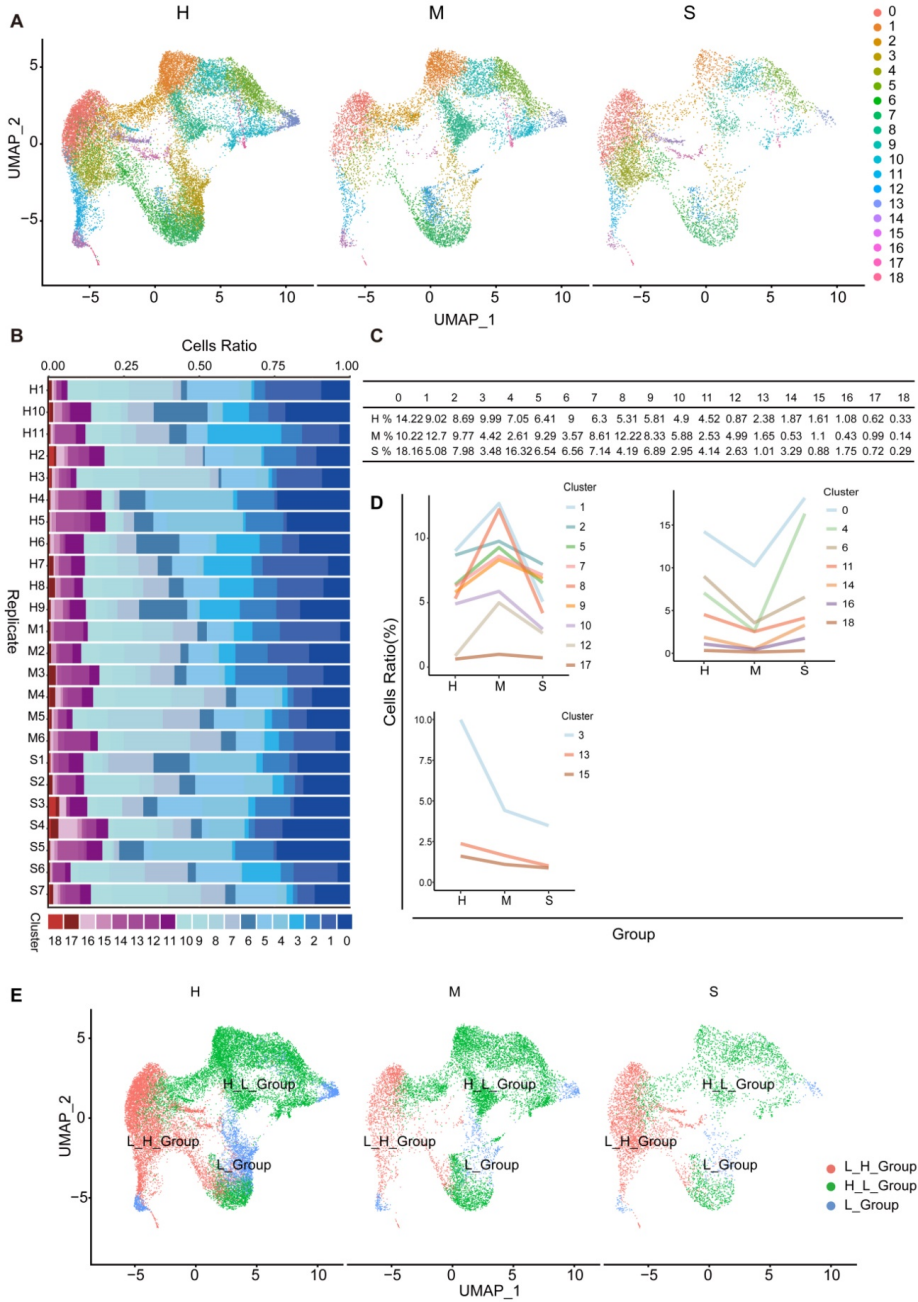
** Subgroup composition and dynamic changes of NK cells.** (A) UMAP projection in NK cell subgroups shows healthy (n = 11), moderate (n = 8), and severe (n = 7) patient samples in different clusters. (B) The bar chart shows the composition of NK cell clusters at the single sample level, colored according to different cluster types. (C) The proportion of each NK cell cluster obtained from healthy (n = 11), moderate (n = 8), and severe (n = 7) patient samples. (D) The line graph shows the changes in the number of healthy (n = 11), moderate (n = 8), and severe (n = 7) patient cells. (E) UMAP projection shows the renaming of NK cell subgroups.

**NK cell subgroups and status in the blood of COVID-19 patients.** To further understand the changes in NK cells in healthy individuals and moderate and severe COVID-19 patients, we used UMAP to divide NK cell subgroups into 19 different clusters (Figure 7A). We counted the changes in the number of cells of these different clusters in healthy individuals, moderate and severe patients, and found that the proportion of cells in the 19 clusters at different stages was different (Figure 7B and 7C). According to the type of response to the virus, we divided them into three different types. In subgroups, the number of cells first from high to low (H_L_Group), first from low to high (L_H_Group), and continuously low (L_Group) (Figure 7D and 7E).

**Figure 8:**
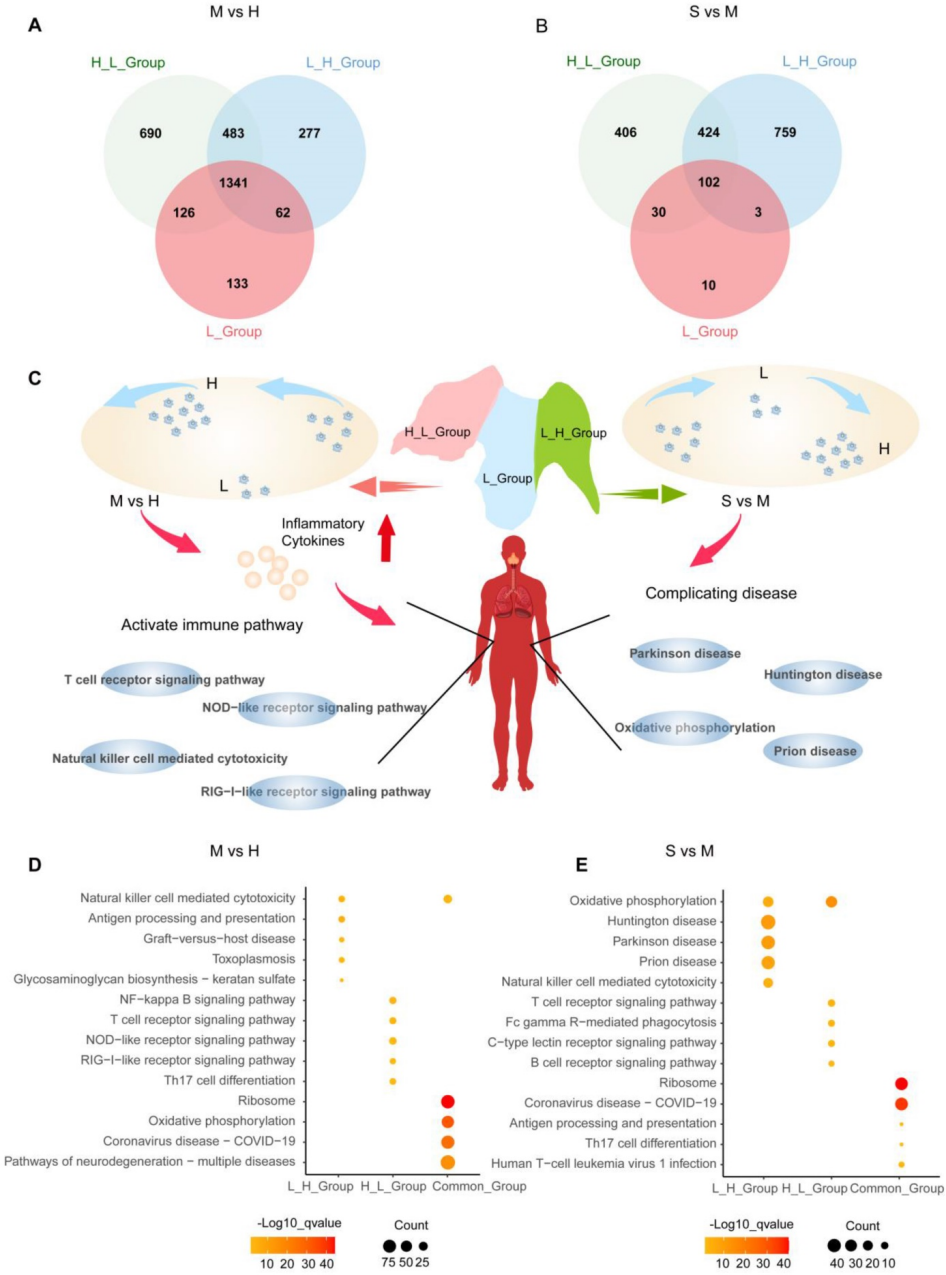
** NK cell response mode during COVID-19 infection.** (A) Moderate COVID-19 patients have a gene overlap in NK cell subgroups (H_L_Group, L_H_Group, and L_Group). (B) Severe COVID-19 patients have a gene overlap in NK cell subgroups (H_L_Group, L_H_Group, and L_Group). (C) Summarize the impact on the H_L_Group and L_H_Group subgroups in the process of COVID-19 infection. (D) In NK cell subgroups, moderate patients (M) were compared with healthy controls (H). H_L_Group specific genes, L_H_Group specific genes, and genes common to all NK cell subgroups (Common_Group) KEGG pathway enrichment analysis, node size represents the number of genes, and color intensity represents -log10 q-value. (E) In NK cell subgroups, severe patients (S) were compared with moderate patients (M). H_L_Group specific genes, L_H_Group specific genes, and genes common to all NK cell subgroups (Common_Group) KEGG pathway enrichment analysis, node size represents the number of genes, and color intensity represents -log10 q-value.

To explore whether the pathways exist heterogeneously in different NK cell subgroups, we intersect the differential genes of each cell subgroup in healthy individuals, moderate and severe patients to find unique genes (Table S6). In healthy individuals progressing to moderate disease, H_L_Group has 690 genes, L_H_Group has 277 genes, L_Group has 133 genes, and there are 1341 genes in Common_Group (Figure 8A). We enriched these genes with GO and KEGG respectively. GO analysis shows that H_L_Group is mainly enriched in regulation of mRNA metabolic process, RNA splicing; L_H_Group is mainly enriched in regulation of DNA-binding transcription factor activity, regulation of response to biotic stimulus; Common_Group is mainly enriched in RNA catabolic process and mRNA catabolic process (Figure S5A-S5C; Table S7).

In addition, KEGG enrichment analysis revealed a moderate stage of SARS-CoV-2 infection, and we found that SARS-CoV-2 infection shares other viral infection pathways, such as Epstein-Barr virus infection, Human cytomegalovirus infection, Hepatitis B, Human papillomavirus infection, Human T-cell leukemia virus 1 infection, Hepatitis C, Measles, Kaposi sarcoma-associated herpesvirus infection, Viral myocarditis, Prion disease. In addition, in our analysis, SARS-CoV-2 infection was found to be the same as bacterial, parasitic, and protozoan disease pathways. Related to Shigellosis, Salmonella infection, Pathogenic Escherichia coli infection, Toxoplasmosis, Vibrio cholerae infection, Leishmaniasis, Legionellosis.

We also found that SARS-CoV-2 has carcinogenic effects, is also closely related to Pathways of neurodegeneration-multiple diseases, and shares pathways with other diseases. Pathways related to SARS-CoV-2 infection also include MAPK signaling pathway, RNA transport, Apoptosis, NOD-like receptor signaling pathway, Thyroid hormone signaling pathway, FOXO signaling pathway, Ubiquitin mediated proteolysis, Spliceosome, Cellular senescence, NF-kappa B signaling pathway, Neurotrophin signaling pathway, Cell cycle, Endocrine resistance, C-type lectin receptor signaling pathway, Th17 cell differentiation, RIG-I-like receptor signaling pathway, Prolactin signaling pathway, Antigen processing and presentation, Natural killer cell mediated cytotoxicity, Glycosaminoglycan biosynthesis - keratan sulfate, Graft-versus-host disease, Ribosome, Thermogenesis, Oxidative phosphorylation, Spliceosome, Endocytosis, Protein processing in endoplasmic reticulum, RNA transport, Phagosome, RNA degradation, mRNA surveillance pathway, Proteasome, Protein export (Figure 8C and 8D; Table S8).

In order to further investigate the role of specific genes in each subgroup of COVID-19 patients as they progressed from moderate to severe, we applied the same method above to obtain 406 genes in H_L_Group and 759 genes in L_H_Group. There were 10 genes in L_Group and 102 genes in Common_Group (Figure 8B). We performed GO and KEGG enrichment analyses on these genes. GO analysis shows that H_L_Group is mainly enriched in oxidative phosphorylation, mitochondrial ATP synthesis coupled electron transport; L_H_Group is mainly enriched in neutrophil mediated immunity, neutrophil activation; Common_Group is mainly enriched in RNA SRP-dependent cotranslational protein targeting to membrane, cotranslational protein targeting to membrane (Figure S5D-S5F; Table S9).

In addition, KEGG enrichment analysis found that H_L_Group was mainly enriched in Pathways of neurodegeneration-multiple diseases, Amyotrophic lateral sclerosis; L_H_Group Mainly enriched in Pathways of neurodegeneration-multiple diseases, Amyotrophic lateral sclerosis; Common_Group was mainly enriched in Coronavirus disease-COVID-19, Ribosome (Figure 8C and 8E; Table S10).

**Figure 9:**
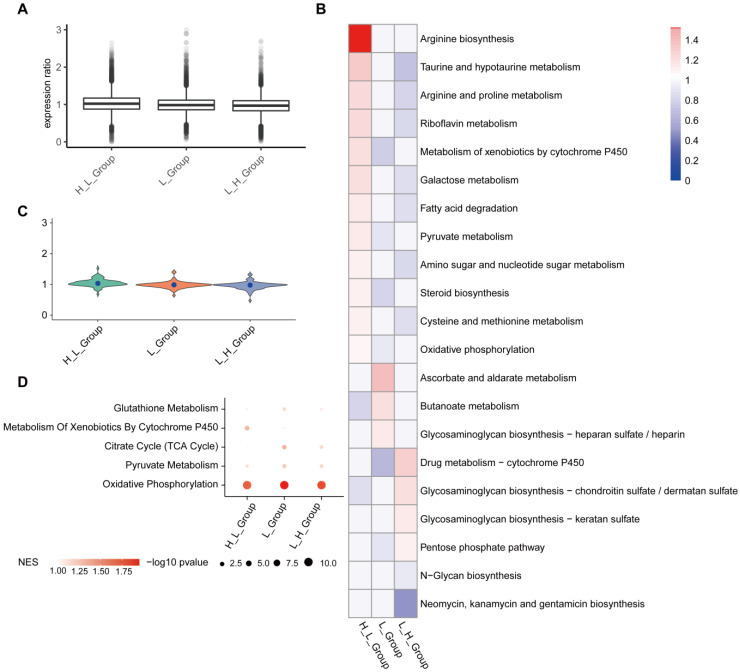
** NK cell subgroups were significantly up-regulated in oxidative phosphorylation.** (A) The Deconvolution method was used to standardize the single-cell data, and the results showed that the expression ratio after the correction was more consistent. (B) Metabolic pathway activity in NK cell subgroups. (C) Distribution of metabolic pathway activity in NK cell subgroups. (D) Major metabolic pathways are enriched in all subgroups. The node size is -log10 p-value. Color intensity indicates the enrichment score (NES) after correction.

In order to obtain the metabolic activity of cell subgroups, we used the single-cell expression data matrix as input data, and we downloaded the metabolic gene set from GSEA (https://www.gsea-msigdb.org/gsea/index.jsp). In addition, we use the scimpute package to assign missing values to the data and used the Deconvolution method to standardize single-cell data (Figure 9A). H_L_Group has high activity in Arginine biosynthesis, Taurine and hypotaurine metabolism; in L_H_Group, Drug metabolism-cytochrome P450, Glycosaminoglycan biosynthesis-chondroitin sulfate / dermatan sulfate has high activity; in L_Group, Ascorbate and aldarate metabolism, Butanoate metabolism had high activity (Figure 9B). On the whole, H_L_Group has higher activity in the entire metabolic activity analysis (Figure 9B and 9C). The results of GSEA showed that NK cell subgroups were significantly up-regulated in oxidative phosphorylation (Figure 9D).

**Figure 10:**
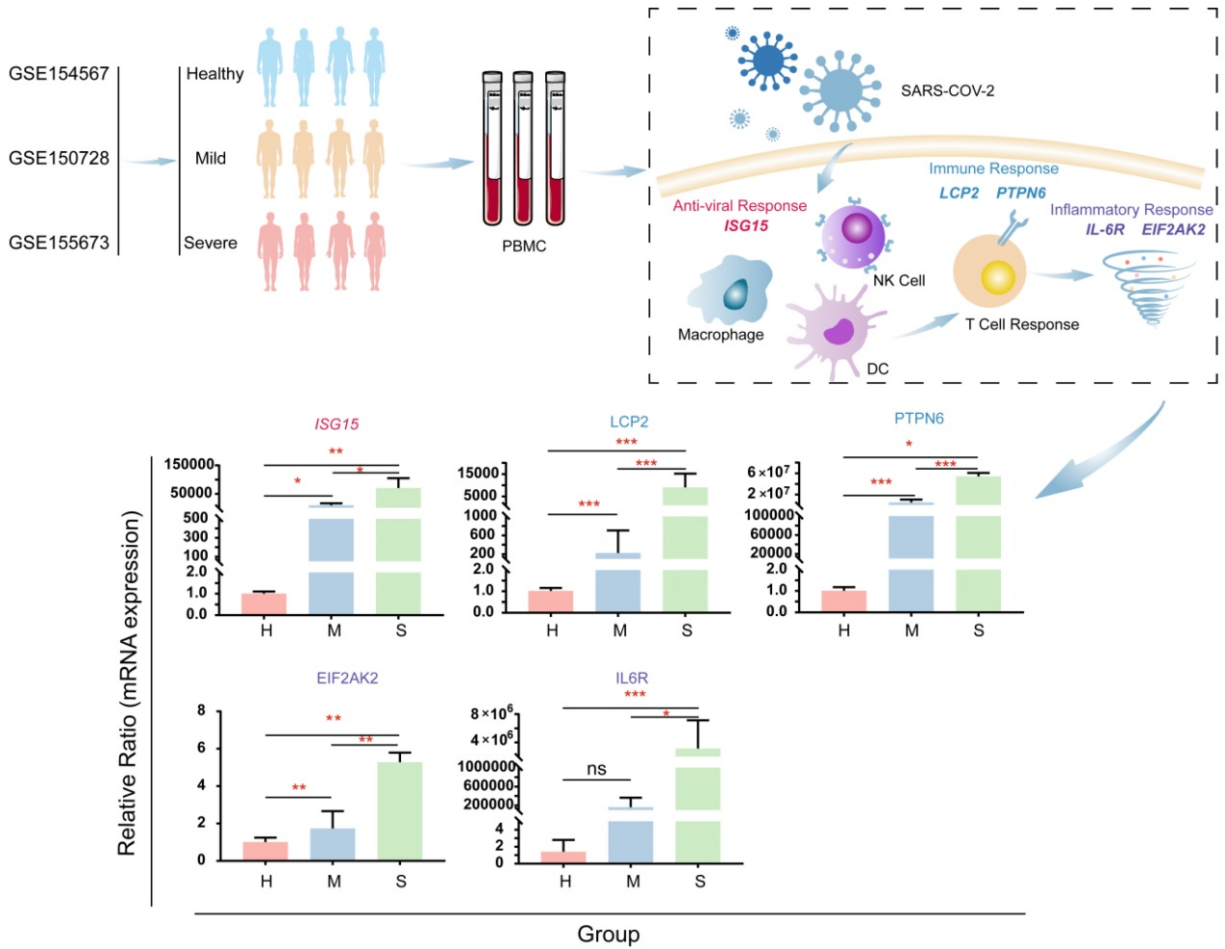
** Validate genes associated with antiviral, immune response, and cell storm during COVID-19 infection.** The real-time quantitative polymerase chain reaction was used to detect COVID-19 in blood samples of moderate patients, severe patients, and healthy people. The expression levels of ISG15 (the core participant involved in the host's antiviral response), LCP2 and PTPN6 (promoting the cell-to-cell spread of viruses, the regulator of antiviral immunity), EIF2AK2 and IL6R (genes related to a strong inflammatory response).

**Experimental detection of T cell and NK cell related pathway genes.** In scRNA-seq analysis, we found that some genes involved in the T cell receptor signaling pathway, Coronavirus disease-COVID-19, and Natural killer cell-mediated cytotoxicity pathway were stably up-regulated during the pathogenesis of COVID-19 patients. To further validate this finding, we performed a quantitative real-time RT-PCR test to detect 11 COVID-19 patients (9 mild patients, 2 severe patients) And the expression of related genes in normal controls (Figure 10). ISG15 is an interferon-inducible protein that is a central player in the host antiviral response, directly inhibiting viral replication and modulating host immunity^12^. During the COVID-19 infection process, its expression is increasing (Figure 10). LCP2 (also known as SLP-76) regulates T cell receptor signaling, and it is also a key determinant of NK cell development and NK cell-mediated elimination of missing self-target cells^13^, ^14^. Studies have shown that SLP-76 plays a key role in promoting the spread of viruses between cells^15^. And our experiments have proved that there is a significant increase in the pathogenesis of COVID-19 (Figure 10). Targeting SLP-76 may be a new target for future therapeutic interventions against COVID-19. PTPN6 (also known as SHP-1) is a negative regulator of antiviral immunity and suggests that SHP-1 may be a target for intervention in acute viral infections^16^. It was found in the experimental results that the expression of this gene increased during COVID-19 infection (Figure 10). In the future, PTPN6 can be used as a molecular target for the impact of COVID-19 infection. EIF2AK2 also increased significantly during the disease (Figure 10). Studies have reported that EIF2AK2 has a broad antiviral spectrum^17^. IL6R expression was increased in the pathogenic process of COVID-19 (Figure 10). This may be related to the synergistic induction of a strong inflammatory response by elevated IL-6, and patients may benefit from treatment with IL-6 or IL-6R antagonists^18^. These results indicate that ISG15, LCP2, PTPN6, EIF2AK2, and IL-6R are candidate marker genes for SARS-CoV-2 infection.

## Discussion

Patients with COVID-19 showed different clinical characteristics during the disease^19^. SARS-CoV-2 infection can activate innate and adaptive immunity^20^. Coordinated responses that work together in innate and adaptive immune cells may lead to rapid control of the virus, while a failed immune response may lead to virus spread, cytokine storms, and high mortality^21^. Lymphocytes are the core component of the immune system to resist virus invasion, especially T cells and NK cells, which play a crucial role in removing viruses^22^. COVID-19 patients can be treated with T cells and memory NK cells^8^, ^23^. With the development of new sequencing technologies, the ability to use next-generation sequencing to analyze single cells provides a better method for studying the diversity and heterogeneity of cells^24^. Cell subgroups are classified based on their effector function and characteristic transcription factor expression^25^, ^26^. So far, many different T cell subgroups have been defined in humans, but there will likely be many more in the future. This single-cell transcriptomic classification is far more than the traditional cell classification. What is the difference?

Because the current evidence supporting the role of T cells in the protection and pathogenesis of COVID-19 is incomplete, and few studies are analyzing the role of T cells in the progression of COVID-19 disease. Understanding the mechanisms of different T cell subgroups is important for the prevention and treatment of SARS-CoV-2. Continuous viral antigen stimulation and immune disorders may lead to T cell failure, a state of T cell dysfunction that occurs during many chronic infections and cancers^27^. We divided the cells into 19 clusters through single-cell analysis (Figure 1C). Compared with traditional methods, this method can separate more cell types, which helps us to have a more detailed understanding of the pathogenesis of T cells in COVID-19. Next, we reclassified four groups according to the type of response to the virus: H_L_Group, L_H_Group, L_Group, and H_Group (Figure 4D and 4E), to explore the function of this new grouping in the infection process of COVID-19 patients. We further expressed through the CD4^+^ marker (CD4, CTLA4, FOXP3, IL2RA) and CD8^+^ marker (CD8A, CD8B, GZMK) and found that CD4^+^ is expressed in H_L_Group and L_H_Group (Figure S3A**)**, but the expression level in H_L_Group is the highest, and CD8^+^ is expressed in H_L_group and L_H_group (Figure S3B**)**. In the process of COVID-19 infection, a large number of CD4^+^ T cells, CD8^+^ T cells, and neutralizing antibody response all contribute to clearing the acute infection^28^. Through KEGG enrichment analysis, we found that H_L_Group mainly activates the immune pathway in the early stage, and is involved in bacterial virus infection. With the increase of viral load, the immune activity decreased. In the later stages of disease development, disease-inducing pathways become activated (Figure 5C). L_H_Group activates pyruvate metabolism, citrate cycle (TCA cycle) in the early stage, and activates oxidative phosphorylation in the later stage of disease development, which is related to metabolic pathways in the process of COVID-19 infection (Figure 5C). It has been reported that the TCA cycle is affected by SARS-CoV-2 infection. One of the most important metabolic pathways^29^.

NK cells play an important role in controlling the early immune response of viral infections^30^. We found that NK cells in the blood of severe COVID-19 patients were exhausted, and the NK cell count was significantly lower than that of mild patients or healthy controls, which is consistent with previous studies^11^, ^31^-^34^. NK cell depletion may be caused by immunosuppression caused by a viral infection, triggering cell apoptosis, or down-regulation of viral cytotoxicity^35^. We further studied the role of NK cell subgroups in the different stages of SARS-CoV-2 infection. According to the type of virus response, we divided NK cells into three types: H_L_Group, L_H_Group, L_Group (Figure 7D and 7E). Through KEGG pathway enrichment, we found that the early stage of viral infection mainly involves viral and bacterial infection and the activation of immune pathways (Figure 8C). In the late stage of viral infection, many disease-inducing pathways are activated (Figure 8C). NK cells are crucial in the early immune response against viral infections, especially by eliminating virus-infected cells^35^. If the drug is actively used in the early stage of SARS-CoV-2 infection, it will reduce the occurrence of complications.

We further analyzed the metabolic activity of immune cell subgroups and found that oxidative phosphorylation dominates the metabolic heterogeneity of T cell and NK cell subgroups (Figure 6 and Figure 9). Oxidative phosphorylation is an important metabolic pathway of SARS-CoV-2 infection, which is consistent with previous studies^36^. Previous studies showed that the virus was changed by the passivation mPTP mitochondrial metabolism, to facilitate its production, while OXPHOS inhibition of viral replication is attenuated^37^, the virus replication and mitochondrial interaction may be necessary for viral load increases^38^. Studies have reported that the virus often changes the function of OXPHOS human main metabolic pathways to provide energy, biosynthetic resources, and immune escape to promote its production. It has now been used as an important target for the treatment of viral infections^39^, ^40^, and immune metabolism can be used as a cell-specific therapy targeting COVID-19^41^. At present, there is limited research on metabolism in COVID-19, and our research provides a new target for the treatment of COVID-19.

In summary, our research is the first to use the type of virus response to analyze blood immune cell subgroups in the course of COVID-19 patients. Compared with the traditional cell classification method, our method can more accurately study the antiviral immune regulation mechanism of COVID-19 patients. Our research provides a new target pathway for the further research of COVID-19, and immune metabolism can be used as SARS-CoV-2 is a new therapeutic target. Understanding these regulatory mechanisms will help develop appropriate diagnostic methods and new therapeutic targets.

## Materials and Methods

**Source data.** Download COVID-19 patient single-cell sequencing data GSE154567, GSE155673, and GSE150728 from Gene Expression Omnibus (GEO) (http://www.ncbi.nlm.nih.gov/geo/). The data set consists of blood single-cell sequencing data of eight moderate patients, seven severe patients, and eleven healthy people.

**scRNA-Seq data processing.** We used the Seurat R package (version 3.2.0) to convert it into a Seurat object for quality control. The following criteria were then applied to each cell of all fifteen patients and eleven healthy controls: more than 15% of UMI cells that had less than 300 expressed genes or derived from the mitochondrial genome were discarded. After filtration, a total of 136,014 cells remained for the following analysis. The genetic barcode matrix of all filtered samples is integrated with Seurat to eliminate batch effects across different individuals. Each Seurat object in the list performs lognormalize standardization and searches for hypervariable genes. The hypervariable genes of all Seurat objects are ranked together, and the top 2000 genes are selected as anchor genes. Each Seurat object uses anchor genes. Perform principal component analysis (PCA) dimensionality reduction, calculate anchor data sets in the PCA space and integrate the data for downstream analysis.

**Single-cell transcriptome recognizes cell types.** Next, cluster analysis is performed on the first 30 principal components, and the clusters are visualized using UMAP to visualize the cells. At the same time, the data based on PCA dimension reduction was clustered to perform clustering analysis using Seurat, and the resolution is set to 0.7 to obtain better results. Determine cell type characteristics based on the expression of known marker genes in the cell marker database^42^. NK cells and T cells were re-clustered using Seurat v.3. In the clustering step, the NK cells and T cells of all samples were re-clustered using the same parameters as above, and the parameter resolution was set to 0.9.

**Identification of differentially expressed genes.** Seurat FindAllMarkers function is used for differential analysis between the control group and disease group of the same cell type. The functional parameters we used in Seurat v.3 are default values. For each cluster, a differentially expressed gene (DEG) relative to all other cells is generated.

**Gene function analysis.** The ClusterProfiler R software package^43^. GO and KEGG enrichment analysis of DEGs can be realized. DEGs with adjusted P value<0.05 were enriched in a large amount. We use bubble charts to show the GO and KEGG pathways enriched in each cell subgroup.

**Cell subgroup metabolism pathway activity and GSEA analysis.** In order to analyze the metabolic pathway activity of cell subgroups, we use the latest algorithm to score the pathway activity of the subgroups^44^. The data we use was the single-cell data expression matrix of cell subgroups, and the count expression data of cell subgroups were converted into TPM, and log conversion was performed as input data. In addition, we entered the GSEA analysis, the software used was javaGSEA (https://www.gsea-msigdb.org/gsea/downloads.jsp), which adopts KEGG_metabolism of data sets.

**Validate genes associated with antiviral, immune response, and cell storm during COVID-19 infection.** We obtained nucleic acid samples from COVID-19 patients (9 mild patients and 2 severe patients) and 2 healthy individuals from the Luzhou Center for Disease Control and Prevention. Next, the FastKing RT Kit (TIANGEN, Chengdu, China) was used for reverse transcription to prepare cDNA. 2 x TSINGKE Master qPCR Mix (SYBR Green I) kit (TSINGKE, Beijing, China) was used for 10 uL quantitative polymerase chain reaction (qPCR). 18sRNA was used as an internal reference. All primers were obtained from TSINGKE (Beijing, China) and are listed in Table 1. mRNA expression levels were determined using Equation 2^-∆∆Ct^.

**Statistical analysis.** Statistical analysis was performed using GraphPad Prism 7.0 software. The student's t-test was used to compare the differences between the two groups, and p<0.05 was considered statistically significant.

## Supplementary Material

Supplementary figures.

Supplementary tables.

## Figures and Tables

**Table 1:** List of primers

Primer name	Forward Sequence (5'-3')	Reverse Sequence (5'-3')
PTPN6	GCCTGGACTGTGACATTGAC	ATGTTCCCGTACTCCGACTC
LCP2	GGAAGAAGCCACCTGTGCCAAA	GCTCATAGGAAGTAGTGCTGGC
ISG15	CTCTGAGCATCCTGGTGAGGAA	AAGGTCAGCCAGAACAGGTCGT
EIF2AK2	GAAGTGGACCTCTACGCTTTGG	TGATGCCATCCCGTAGGTCTGT
IL6R	GACTGTGCACTTGCTGGTGGAT	ACTTCCTCACCAAGAGCACAGC
18sRNA	AAGTCCCTGCCCTTTGTACACA	GATCCGAGGGCCTCACTAAAC
