# Supplementary material for: Dynamic response landscape of immune cells identified immune dysfunction which predicts disease progression in COVID-19 infected patients
Source: Int J Biol Sci. 2022 Apr 24;18(7):3066–81. doi: 10.7150/ijbs.71163 (PMC9066099; doi:10.7150/ijbs.71163)
Supplement: Supplementary file 1 — Supplementary figures. [file ijbsv18p3066s1.pdf]

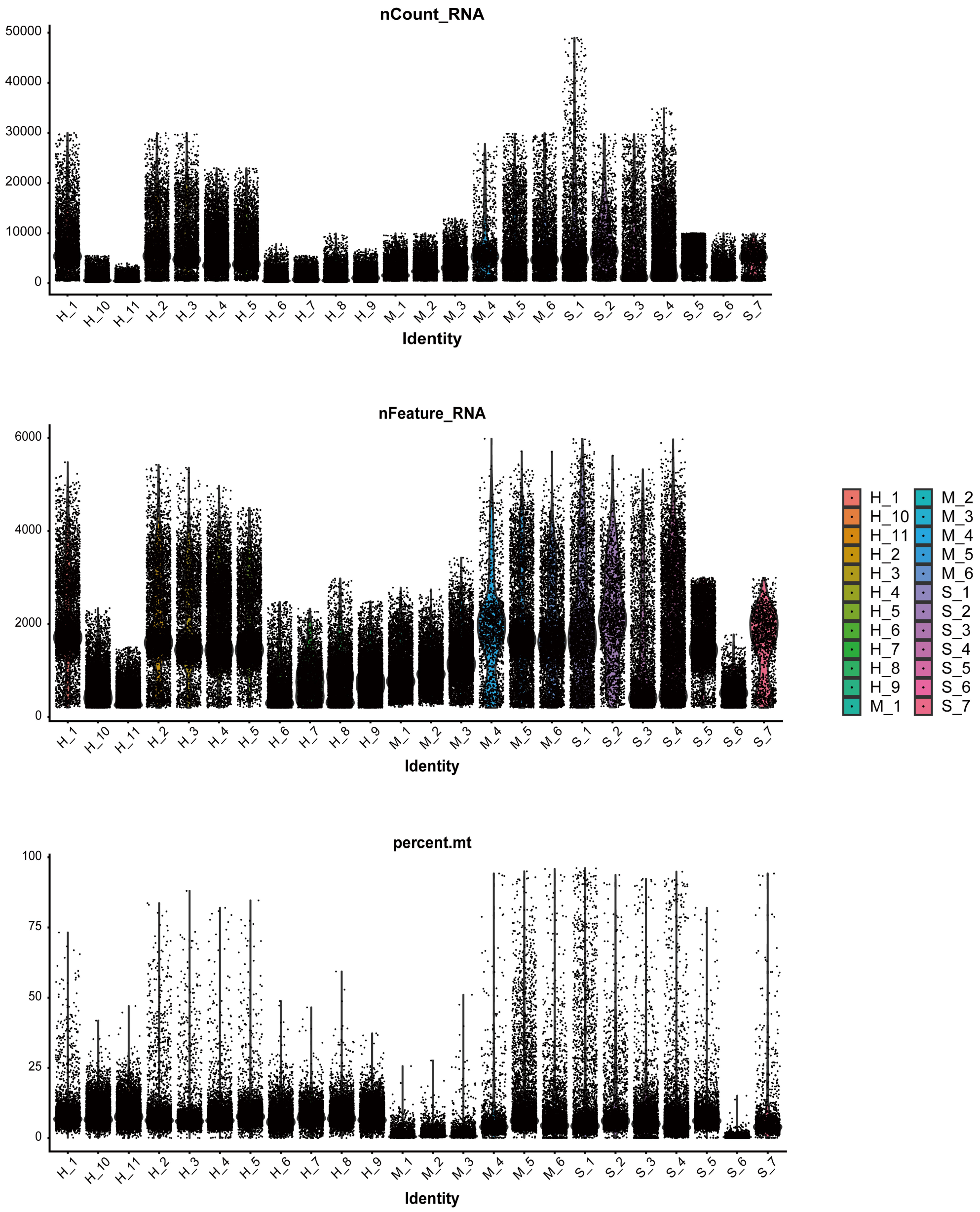

Figure S1

Quality control of single-cell data of PBMC samples from COVID-19 patients. Violin plot displays cell quality control index information, including the number of genes detected in each sample cell, the unique molecular identifiers (UMIs) detected in the cells, and the proportion of mitochondrial genes.

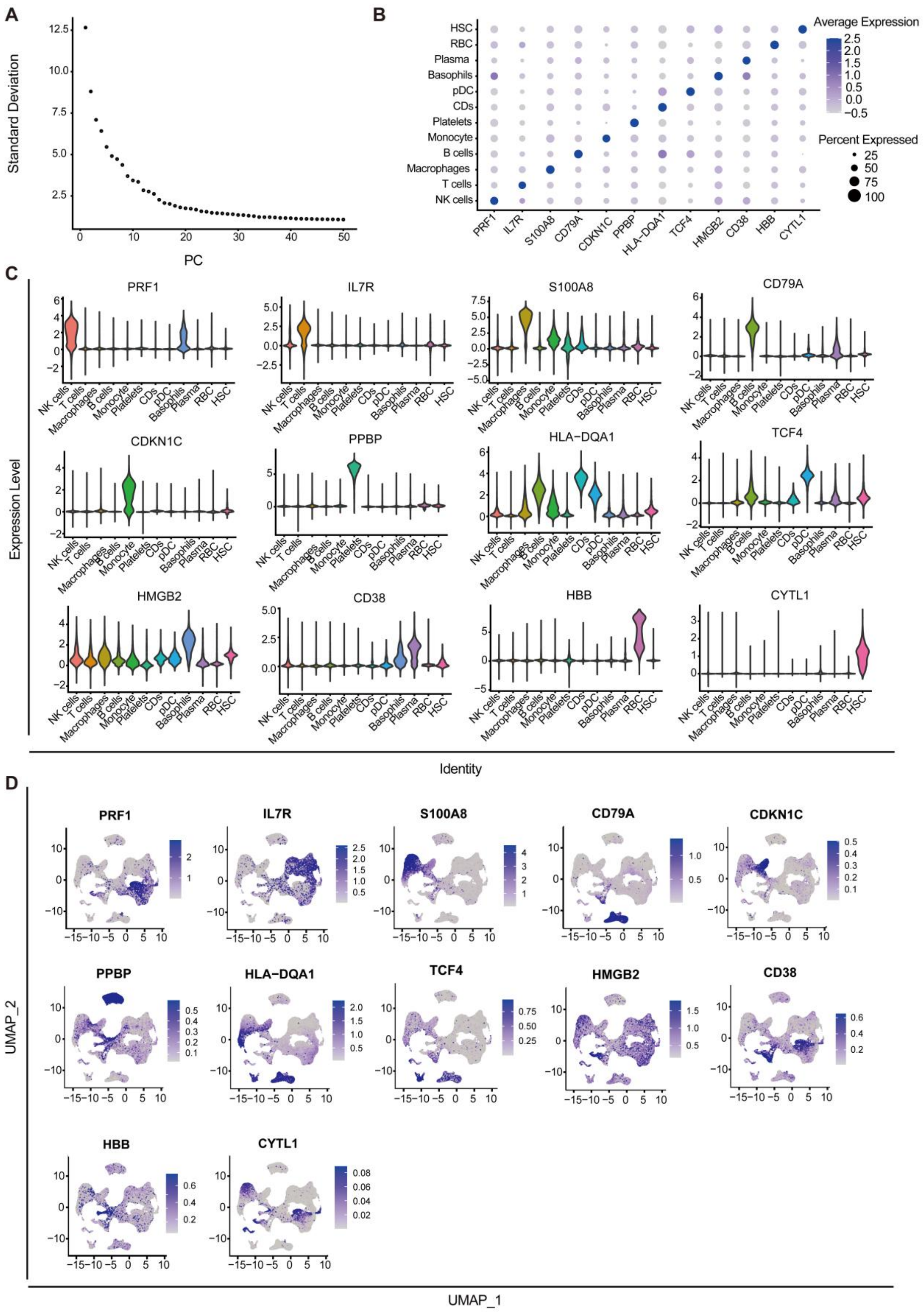

Figure S2

Expression levels of key genes in PBMC cells of COVID-19 patients. (A) ElbowPlot to determine the dimensionality of the data set. The top 30 PCs can explain most of the variation. (B-D) Bubble plots, violin plots, and UMAP plots show the

expression levels of marker genes in 12 cell types.

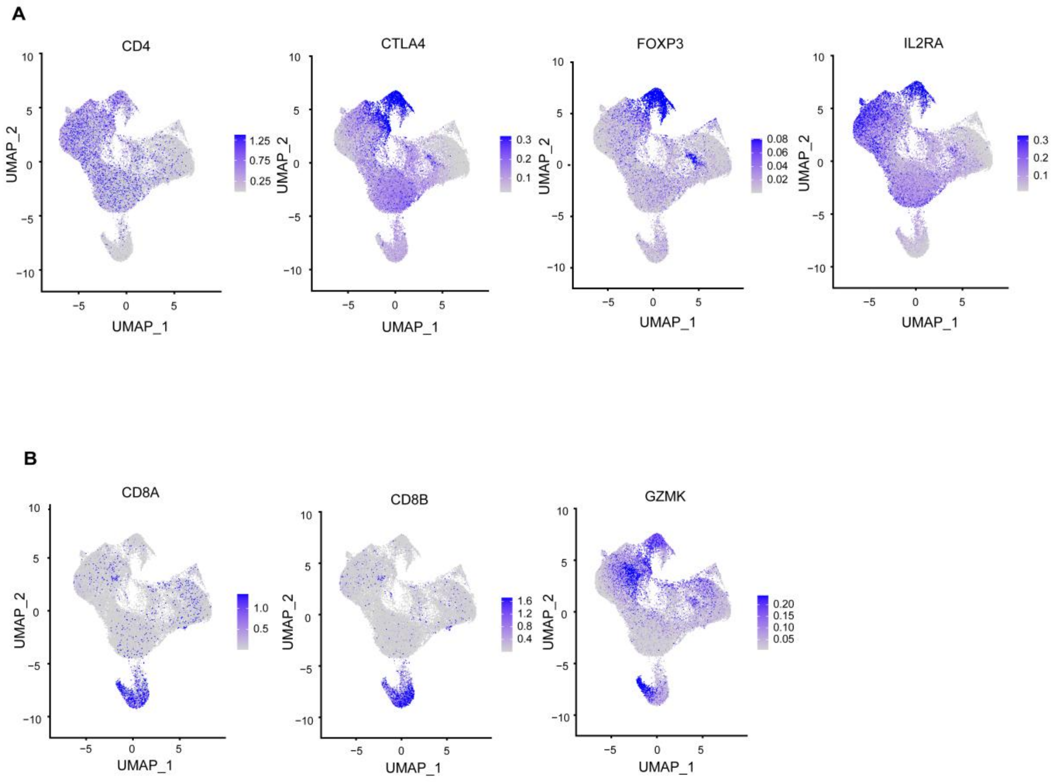

Figure S3

Expression levels of key genes in CD4+T cells and CD8+T cells. (A) UMAP projects the expression of CD4+ T cell marker genes in T cell subgroups. (B) UMAP projects the expression of CD8+ T cell marker genes in T cell subgroups.

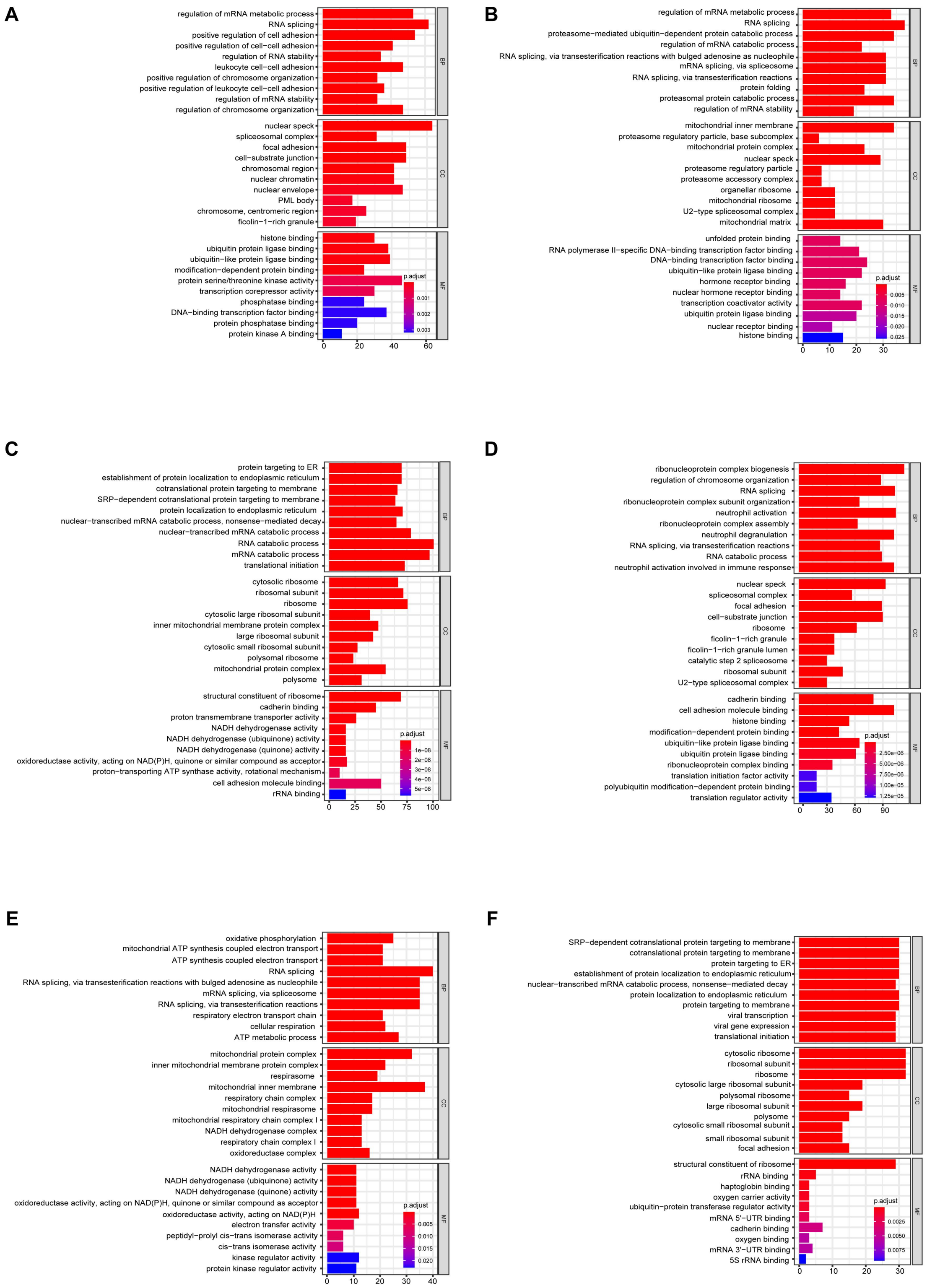

Figure S4

GO enrichment analysis of T cell subgroups. (A-C) GO enrichment analysis of H_L_Group, L_H_Group, and Common_Group in COVID-19 patients progressing from healthy to moderate disease. (D-F) COVID-19 patients develop from moderate to severe disease, GO enrichment analysis of H_L_Group, L_H_Group, and

Common_Group.

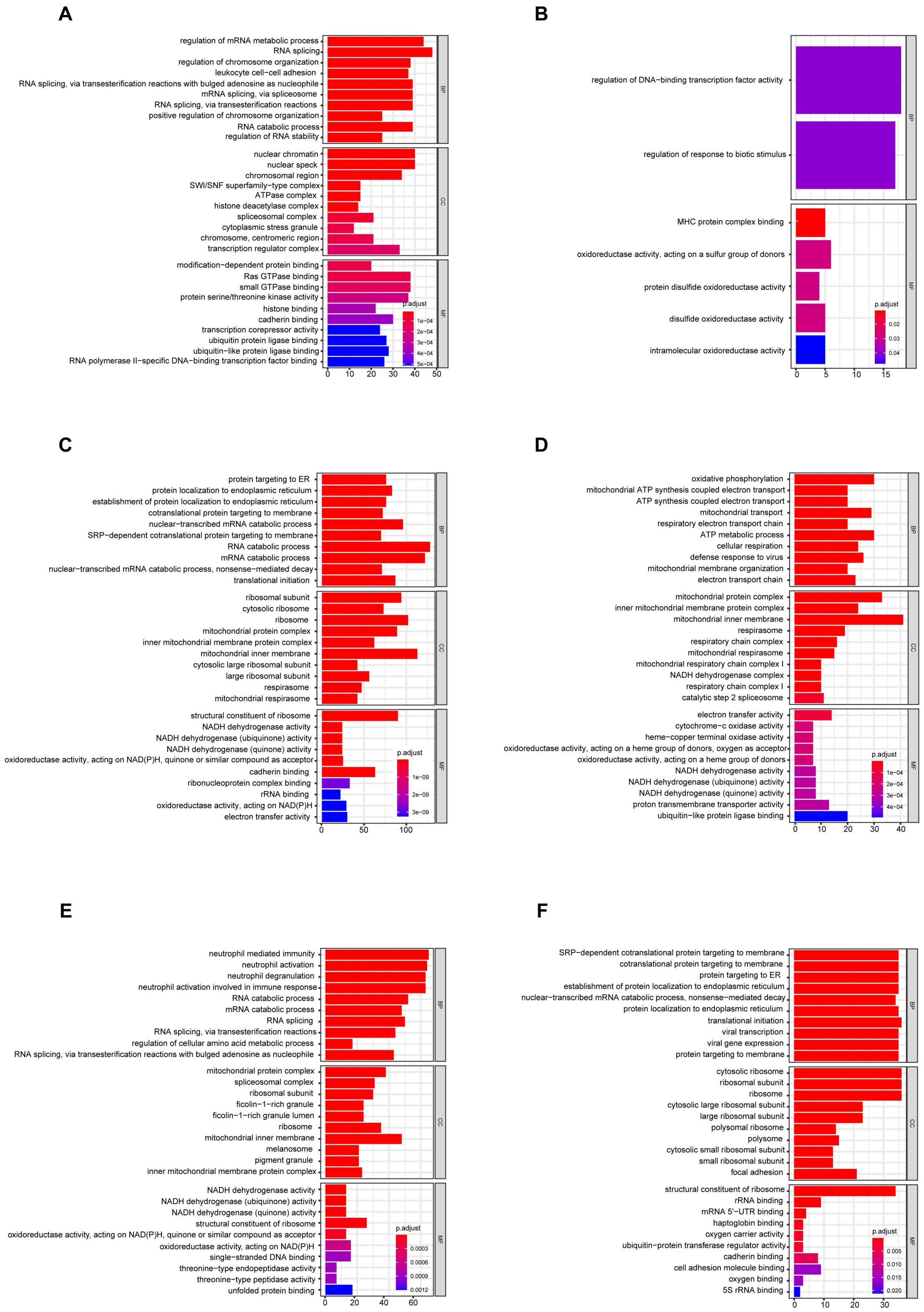

Figure S5

GO enrichment analysis of NK cell subgroups. (A-C) GO enrichment analysis of H_L_Group, L_H_Group, and Common_Group in COVID-19 patients progressing from healthy to moderate disease. (D-F) COVID-19 patients develop from moderate

to severe disease, GO enrichment analysis of H_L_Group, L_H_Group, and Common_Group.
